# Understanding and navigating the repercussions of the politically polarized climate in mental health

**DOI:** 10.47626/2237-6089-2021-0350

**Published:** 2023-06-27

**Authors:** Elisa Brietzke

**Affiliations:** 1 Department of Psychiatry Queen’s University School of Medicine Kingston ON Canada Department of Psychiatry, Queen’s University School of Medicine, Kingston, ON, Canada.; 2 Centre for Neuroscience Studies Queen’s University Kingston ON Canada Centre for Neuroscience Studies (CNS), Queen’s University, Kingston, ON, Canada.

**Keywords:** Psychotherapy, psychosocial model, psychiatric care

## Abstract

The world is experiencing a moment of political polarization between liberal and conservative ideas, which has aggravated since the arrival of the Covid-19. Many countries (Brazil included) have been experiencing the generalized occurrence of people fighting over politics, in contexts including family, workplace, friendships, and romantic relationships. Over the past 2 years, it has been possible to observe an unexpected and overwhelming effect of the political climate on psychotherapy patients, some of whom have started to actively look for therapists who share their convictions. Brazil is experiencing a moment of severe sanitary, economic, social, and political crisis, which is directly affecting our patients. Nevertheless, the impact of the political climate on our population has not been systematically investigated. However, as the political environment is an inherent part of the social component of the psychosocial model, it is important that mental health professionals be prepared to have this conversation with their patients. This highlights the need to address these difficulties in supervision, rounds, and clinical discussions.

It is no secret that the world is experiencing a moment of political polarization between liberal and conservative or ultra-conservative ideas. The remarkable increase in the attention that extreme ideas attract from a general audience together with the expansion of social media use have been recognized as major contributors to this environment of animosity.^1^ For example, a content analysis of more than 2 million posts from Twitter and Facebook found that posts aggressively criticizing ideas or individuals from opposite groups generated much more engagement than neutral or less aggressive posts.^1^

The arrival of the coronavirus in North America, South America and Europe found an already divided society and political ideologies shaped perceptions of the pandemic, including adherence to protective measures such as mask wearing and vaccines.^2^ For example, one study conducted in the United States in 2020 found that conservatives were more likely to state that the pandemic was receiving too much media coverage and that people were overreacting to the virus.^3^ On the other hand, liberals tended to report that government was not responding to Covid-19 with the necessary vigor. In this study, social distancing behaviors were more common among liberals and were associated with more depressive symptoms.^3^ The understanding that deaths, financial losses, and other consequences of Covid-19 were directly derived from political choices aggravated these conflicts.

Lockdown measures and stay at home orders forced most people to spend more time online and a dramatic shift took place in the way we use the internet. The traffic to traditional media vehicles largely increased and the same happened with Wikipedia, and reliable websites with information about Covid-19, such as the websites of the Centers for Disease Control and the Johns Hopkins Coronavirus Research Centre.^4^ However, other internet use phenomena during this period include massive increases in access to videogames and pornography. In this melting pot of new available internet content mixed with a remarkable increase in time spent online, it was possible to observe an increase in the circulation of ‘fake news’ and disinformation, some supporting radicalized perceptions of the situation.^5^

Many countries (Brazil included) have been experiencing another pandemic, the generalized occurrence of people fighting with family members or friends over politics. Mental health professionals from different countries are often hearing from their patients that they are frustrated, sad, or angry with people from their circles who support or don’t support a president or who do not share their political preferences.^6^

These conflicts extend beyond families and close friendship circles and also affect workplace relationships. A recent survey conducted by the Society for Human Resource Management (SHRM) has shown that 42% of Americans experienced political disagreements with coworkers, which resulted in stress and experiences of discrimination at the workplace due to political opinions.^7^ Finally, political disagreements are also affecting romantic relationships. One of the most popular dating apps found a 187% increase in mentions of political affiliations in its chats.^8^ Dating platforms report that between 70 and 84% of their members are unwilling to date someone with opposing political views and most of these platforms have developed filters to allow their users to choose not to see other members who do not share their views.^8^

One of the recent changes in the political environment is the perception that political differences are not just simply based on opinions about taxes, economic measures, or ideological views, but that they are strongly rooted in people’s characters and personalities. For example, a poll conducted by the Public Religion Research Institute during the recent American election found that around 80% of respondents who self-identified as Republicans believed that the Democrat Party had been taken over by Socialists and that around 80% of respondents who self-identified as Democrats believed that Republicans are racists.^9^ Taking these findings together, it is natural to expect that these conflicts would arrive in mental health settings, including psychiatric and psychotherapy services, requiring understanding and reflection on this topic from professionals.

One of the characteristics of this political polarization is the relative impermeability of a relatively large proportion of the population to opposite ideas, with some individuals even showing a very dogmatic (and sometimes religious) view of reality. Specifically in Brazil, where the Covid-19 situation unfolded in particularly severe ways, with more than 500,000 deaths so far, some individuals actively ask about the political views of their doctors before seeking help.^10^ This unexpected search for information about the political views of professionals has also arrived in mental health care.

Solomon and Barber^11^ reported that over the past 2 years it has been possible to observe an unexpected and overwhelming effect of the political climate in psychotherapy patients, with these discussions taking center stage in multiple sessions, in a way that has not been observed since 9/11. A recent study involving a sample of 604 Democrat and Republican patients found that two thirds of patients reported engaging in discussions about politics with their psychotherapists and most of them reported perceptions of a better quality therapeutic alliance when they thought the therapist shared the same political views.^12^ The same study shows that 87% of therapists discussed politics with their patients in sessions and that 63% of them reported some degree of self-disclosure of their political views, which happened more frequently when they perceived their patients as sharing their own political views.^11^

Brazil is experiencing a moment of severe sanitary, economic, social, and political crisis, which is directly affecting our patients. Nevertheless, the impact of the political climate in our population has not been systematically investigated. However, as the political environment is an inherent part of the social component of the psychosocial model, it is important that mental health professionals be prepared to have this conversation with their patients. Finally, the issues approached here have implications not only for independent psychotherapists but also for training and supervision.^11^ Psychiatrists and psychotherapists are not immune to the political climate and can experience challenges in management of their own contra-transference. This highlights the need to address these difficulties in supervision, rounds, and clinical discussions.^11^
